# *Corallorhiza striata* is the first example of a pseudocopulatory orchid in North America and an instance of “double deception” in fully mycoheterotrophic plants

**DOI:** 10.1002/ajb2.70185

**Published:** 2026-04-09

**Authors:** John V. Freudenstein, Craig F. Barrett

**Affiliations:** ^1^ Ohio State University Herbarium, Department of Evolution, Ecology and Organismal Biology, The Ohio State University 1315 Kinnear Road Columbus 43214 OH USA; ^2^ Department of Biology West Virginia University 53 Campus Drive Morgantown 26506 WV USA

**Keywords:** Corallorhiza striata, deception, fungi, Ichneumonidae, mycoheterotrophy, Orchidaceae, pollination

## Abstract

**Premise:**

Orchids have many pollination strategies, from highly species‐specific mutualisms with insects to deceit pollination, including sexual deception. The family also has the most leafless, parasitic species (mycoheterotrophs) of any plant family. The occurrence of two types of deception simultaneously in individuals of a single species previously has been suggested to be highly improbable.

**Methods:**

We studied *Corallorhiza striata* in the field, documenting the behavior of pollinators on the plants. We compared structural features of *C. striata* flowers to those of other species in the genus and tested for the presence of nectar. We also examined over 6000 iNaturalist photo records of *C. striata* to expand our pollinator search.

**Results:**

The only pollinators for which we found clear evidence were males of the ichneumonid wasp *Pimpla pedalis*, which visited the nectarless flowers and exhibited mating‐type behavior. The floral structure of *C. striata*, especially with respect to the labellum, is distinct from that of bee‐ and fly‐pollinated species of *Corallorhiza*. We demonstrated that wasps were attracted to flowers in the absence of visual cues.

**Conclusions:**

*Corallorhiza striata* is the first example of a pseudocopulatory orchid from North America, and *Corallorhiza* is only the second genus known to deceive an ichneumonid. It is trophically and reproductively deceptive in the same life stage. Four other fully mycoheterotrophic species and three partial mycoheterotrophs also are known to engage in both deceptions indicating that, although rare, simultaneous deception can be a successful strategy. Such parasitic plants provide opportunities to study unusual evolutionary and ecological phenomena.

Organismal interactions, along with the abiotic influences of their environment, are key to shaping the evolutionary trajectory of any species. Two classes of interactions believed to have been especially important in the success of land plants on the one hand, and within angiosperms on the other, are fungal interactions and insect pollination, respectively (Parniske, 2008; Peris and Condamine, 2024). In both cases, these are fundamentally mutualisms, with fungi providing nutrients and water in exchange for fixed carbon and pollinators receiving a reward of varying type (often nutrition) as inducement to move pollen among individuals of a species. Orchids are no exception to the rule of interaction with both fungi and insects, but proportionally perhaps more than any other large angiosperm clade, they take advantage of these relationships such that partners often receive no benefit for their services, thus wasting the partner's resources invested in the interaction.

With respect to pollination, some orchids are well known for their specialized systems that are mutualistic and rewarding. These include highly species‐specific relationships such as the famous “Darwin's orchid” (*Angraecum sesquipedale* Thouars) and its sphinx moth pollinator, *Xanthopan morganii* Walker (Wasserthal, 1997), the critical limiting features of which are the length of the floral spur and of the insect's proboscis. The euglossine bee relationship, primarily with neotropical Cymbidieae, is focused on floral chemical rewards that are non‐nutritional but have a role in insect behavior. However, in other cases, known as deceit pollination, insects are lured to flowers with an expectation of reward, but none is given (Lunau and Wester, 2017). Far from rare in orchids, deceptive species are estimated to comprise one third to one half of the family (Shrestha et al., 2020; Ackerman et al., 2023), and most deceptive‐pollinated angiosperms are orchids (Renner, 2006). In some cases, orchid flowers mimic other species that provide food rewards but provide none of their own, such as *Disa nivea* H.P. Linder mimicking *Zaluzianskya microsiphon* K. Schum. (Anderson et al., 2005). In other cases, such as *Calopogon* species, labellum structures that mimic pollen‐laden anthers suggest a specific nutritional source that is non‐existent. Perhaps the most striking of the deceit‐pollination systems in angiosperms are those in which male insects are attracted to flowers with the expectation of mating due to visual, olfactory and tactile cues that mimic a female insect. Currently known from only two other angiosperm families (Asteraceae, Ellis and Johnson, 2010; Iridaceae, Vereecken et al., 2012), the great majority of such pseudocopulatory species are orchids (Gaskett, 2011). Even among orchids, pseudocopulatory pollination is rare, traditionally known mostly in members of Australian Orchidoideae.

Turning to fungal interactions with plants, the most widespread and pleisomorphic type of mycorrhiza involves land plants and a member of the fungal group Glomeromycota, resulting in an arbuscular mycorrhizal (AM) interaction that is mutualistic (Smith and Read, 2008). Although orchids associate with fungi, they do not form AM associations. Instead, orchids require a fungal associate for germination at least, and the fungus is not provided a reward as far as currently known, but rather is consumed. In this sense, all orchids are initially mycoheterotrophic (Rasmussen, 1995). In at least 30 separate lineages (Freudenstein and Barrett, 2010), orchids have come to depend fully on fungi for fixed carbon, having lost photosynthesis and leaf laminae, and are known as full mycoheterotrophs or holomycotrophs (Rasmussen, 1995). Other orchid species are photosynthetic but also continue to obtain some of their fixed carbon from fungi and are known as partial mycoheterotrophs or mixotrophs.

A species that derives a benefit from another at a cost to the partner is often termed a parasite and usually exhibits specializations for the relationship. Over the past few decades, such species have gone from being viewed as “evolutionary dead‐ends” and “degenerate forms” (Mayr, 1963; Noble and Noble, 1976) to being appreciated for their ubiquity and importance as components and drivers of biodiversity, shaping character evolution and community structure (Mouritsen and Poulin, 2005; Dunne et al., 2013). Estimates of the number of extant parasitic species vary, but some studies suggest it could be equal to or greater than the number of free‐living species (Price, 1977; Dobson et al., 2008; Larsen et al., 2017). Within seed plants, trophically defined parasites are a clear minority of species (approximately 5000 of an estimated 420,000; Heide‐Jørgensen, 2008), and this role has arisen many times. Such plants either parasitize other plants via haustorial connections (92% of plant parasites) or parasitize fungi (mycoheterotrophy; 8%). Leafless heterotrophic orchids, including *Corallorhiza*, are commonly viewed as parasites of fungi because they derive their fixed carbon (or at least the great majority of it) from them with no evident benefit to the fungi.

Most definitions of parasitism focus on the cost to a host with respect to trophic resources (e.g., Parmentier and Michel, 2013), probably because these are most common. Some parasites, especially unicellular species, also obtain a living environment (e.g., inside the host) via their dependency. Such definitions may thus also emphasize physical contact between parasite and host. However, there are other possibilities for resource targeting relating to reproduction. Brood parasitism is a well‐known phenomenon in which birds and some other animals facilitate care of their young through deception and behavioral manipulation of other species. Although there is a trophic component in this case (feeding of the young), the parasitism is broader, achieving care of progeny and ultimately the parasite's overall success in reproduction at the cost of the host's own progeny (Miller, 1946). In this case, there is no necessary physical contact between the organisms and it does not continue throughout the partners’ lifetimes. It is not our purpose to argue the definition of parasitism here, but we suggest that a broad one is most consistent with the ever‐changing landscape of our knowledge of organismal interactions, especially in light of the context‐dependent, continuum nature of some (Rogalski et al., 2021). We will use the term deception here, recognizing that deceptive relationships can fit into at least some definitions of parasitism, being nonrewarding and coming at a cost. Indeed, deceit pollination has been called flower parasitism by van der Cingel (2001).

Here we present evidence that at least one variety of the North American leafless *Corallorhiza striata* Lindl. (Orchidaceae), targeting a particular species of ichneumonid wasp as pollinator, is the most recently discovered instance of a sexually deceptive species, the first known from North America and making *Corallorhiza* only the second genus found to deceive ichneumonid wasps. Perhaps even more importantly, because it is also a highly specific trophic “cheater” on a mycorrhizal mutualism between a fungus and another plant, *C. striata* is a rare instance of a species known to target two species for deception in a single life stage. It is in this sense a “double deceiver” and one of only a few to be shown to do this—all are leafless mycoheterotrophs. Such a scenario previously had been suggested to be highly improbable (Bidartondo, 2005) but clearly can occur; we discuss the implications of this situation for our understanding of deception.

## MATERIALS AND METHODS

Field observations of *C. striata* var. *striata* and pollinators were made at various sites along the Niagara escarpment in Michigan, United States, during May–June of 2022–2025, where *C. striata* can be locally frequent on soils underlain by limestone. In addition, we collected pollinators in Michigan, Montana, New Mexico, and New York in 1989 and from 2007 to 2009 (Table 1). Video of insects visiting flowers was captured using a remotely triggered Sony A7RIII camera with a 90 mm lens. Additionally, all photographic records of *C. striata* on iNaturalist.org posted through July 2025 were examined (over 6000).

**Table 1:** Localities at which *Pimpla pedalis* was observed visiting *C. striata*. Insect specimens are deposited in the Triplehorn Insect Collection at The Ohio State University except for the Emmet Co., Michigan specimen, which is at the Cornell University Insect Collection. Specimens with * have pollinaria attached.

Locality	Date
Emmet Co., Michigan	13 June 1989
Presque Isle Co., Michigan	11 June 2005*
Schoolcraft Co., Michigan	11 June 2005
Sweet Grass Co., Montana	11 June 2007
Otero Co., New Mexico	15 June 2008
Madison Co., New York	11 June 2009
Mackinac Co., Michigan	8 June 2022
Chippewa Co., Michigan	9 June 2022* (also observed 2024, 2025)

Wasps were captured visiting *C. striata* racemes using an insect net, and sex was determined by checking for the presence/absence of a prominent ovipositor. To determine whether an olfactory cue was active in the absence of visual cues, five racemes of fresh *C. striata* flowers were placed in a 4‐L zip‐closure plastic bag and stored at ambient temperature in the field for 20 min. The plastic bag was placed inside a narrow, ca. 45‐cm‐long cone of double‐thickness black plastic mesh to obscure the flowers. The cone was taken to a clearing near where *C. striata* were growing and the plastic bag opened in the center third of the plastic cone and behavior of any wasps outside the cone was observed.

Solvent extracts of floral fragrance were prepared by placing approximately 20 flowers in 10 mL of 100% HPLC‐grade methanol and another set of 20 in 10 mL of 100% hexane for 1 h at ambient temperature, then the flowers were removed. Target tests were performed in the same clearing where the black cone test was performed. Three bamboo stakes ca. 40 cm long were used as the support for a 2‐cm^2^ piece of black felt that was clipped to the top; the stake was then anchored in the ground; 50 µL of each of the methanol extract, the hexane extract, and a methanol blank were spotted on felt pieces and observed for 30 min, then another 100 µL of either an extract or blank were spotted on the respective pieces of felt, and any wasp visits recorded for the next 15 min.

To test for the presence of nectar in flowers, we used glass capillary tubes (0.5 mm outside diameter) to probe the base of the column and regions where perianth parts attached to the base of the flower, and any fluid uptake was noted. Thirty flowers from multiple plants being visited by wasps were probed at a Chippewa County, Michigan site (near the DeTour State Forest Campground) in 2025.

We compared the structure and function of *C. striata* flowers with those of other members of the genus that are known to be pollinated by nectar‐seeking insects to determine whether there are distinctive specializations in *C. striata*. We used the results of our previous phylogenetic analyses of *Corallorhiza* to guide the structural comparison. *Corallorhiza* comprises two sister clades: the *C. trifida* Chat. group [including also C. *bulbosa* A. Rich. & Galeotti, *C. maculata* (Raf.) Raf., *C. macrantha* Schltr., *C. mertensiana* Bong., *C. odontorhiza* (Willd.) Poir., and *C. wisteriana* Conrad], and the *C. striata* group (including also *C. involuta* Greenm. and *C. bentleyi* Freudenst.) (Freudenstein and Senyo, 2008; Barrett et al., 2014). *Corallorhiza striata* was recently shown to comprise four genetically distinct, allopatric to parapatric lineages that are at least somewhat distinct morphologically, two of which are named varieties (Barrett et al., 2022). The focus here is principally on *C. striata* var. *striata*, but we considered whether the structural features described for that variety also apply to the other varieties of *C. striata* and to *C. involuta* and *C. bentleyi*. The morphology of freshly collected, liquid‐preserved, or dried specimens, including over 10,000 of the latter examined by Freudenstein (1997), was observed for *C. striata* and other members of the genus.

## RESULTS

### Pollinator observations

**Figure 1:**
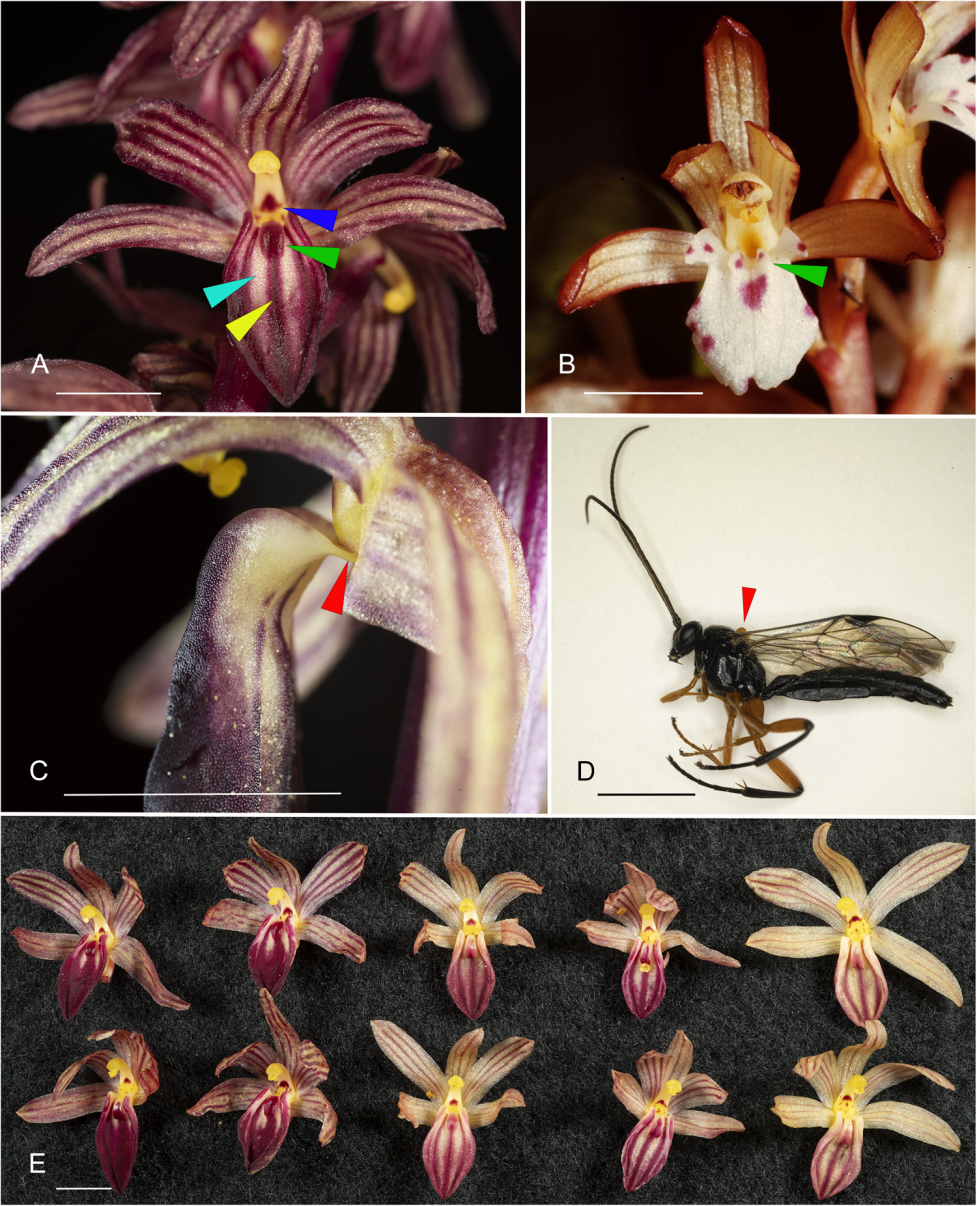
(A) *Corallorhiza striata* var. *striata* flower, front view. Dark blue arrow: red spot at base of column; green arrow: partially fused lip lamellae; light blue arrow: pale patch; yellow arrow: red median stripe. (B) *Corallorhiza maculata* var. *occidentalis* (Lindl.) Ames, front view. Green arrow: free lip lamellae. (C) *Corallorhiza striata* var. *striata* flower, side view. Red arrow: hinge attachment of labellum to base of column. (D) *Pimpla pedalis*, side view. Red arrow: pollinarium attached to mesosoma. (E) *Corallorhiza striata* var. *striata* flowers. Each vertical pair is from a single plant, showing color variation and consistent presence of red spot at base of column. Bar = 5 mm.

All insects we observed visiting *C. striata* var. *striata* in over 20 years of observations of this orchid were individuals of a single ichneumonid wasp species, *Pimpla pedalis* Cresson. Specifically, of the 28 wasps that we captured and sexed individually during this period from Michigan, New York, and Montana, all were male. An additional 13 males were observed in photographs where sex was determinable, making a total of 41 male wasps and no females. We also collected *P. pedalis* visiting flowers of *C. striata* var. *vreelandii* L.G. Williams in New Mexico. Specimens we collected are listed in Table 1 and are deposited in the entomology collections at Cornell University and The Ohio State University. Some observations were the result of fieldwork not focused specifically on pollinators, but more pollinator‐focused observation periods between 2022 and 2025 comprised approximately 22 h. Two of the wasps collected for permanent specimens had pollinaria attached (Figure 1D); we observed additional wasps with pollinaria attached that were not collected. However, not all visits to flowers resulted in removal of pollinaria.

Our video of a wasp visit at one of the Michigan localities shows details of the insect's behavior (Appendix S1). In the video, a male wasp flies to the plant, lands on it and proceeds to move to a flower, approaching and entering it such that it is positioned on the labellum, with its head in proximity to the column. The insect pauses and curves its metasoma (abdomen) forward, then slowly straightens it and flies away.

The results of the visual cue‐obscuring experiment were informative. Within 2 min of opening the plastic bag containing racemes under the center of the back netting cone, a swarm of ca. 10 wasps appeared and focused specifically on this area of the cone, flying around it and landing on it.

Targets loaded with floral extracts also yielded results. The methanol floral extract attracted three wasps to the felt target, either landing on it or hovering close to it. No wasps approached the target with the methanol blank, nor did they approach the one with the hexane extract. Notably, the wasps visited the methanol extract target soon after the extract was applied (within a few minutes), and no more approached after that, suggesting that the compound(s) to which they were responding were volatile and dissipated relatively quickly.

Of the 30 flowers that were probed for nectar with capillary tubes, none yielded any fluid.

### iNaturalist records

Our examination of over 6000 photographically documented observations of *C. striata* on iNaturalist (https://www.inaturalist.org) identified 11 listings with photographs that appear to have the same wasp species visiting the flowers, including two with pollinaria clearly attached to them (iNaturalist 62551: San Mateo Co., California; 219639138: Division No. 6, Alberta, Canada). The localities of the other reports ranged from Ontario to California: Cass Co., Minnesota (47159977); Bruce Co., Ontario (27802065); Division No. 1, Alberta (294605805); Marin Co., California (217784457); Santa Clara Co., California (10477370, 212964564); San Mateo Co., California (217368850); Lane Co., Oregon (132472279); San Juan Co., Washington (122505491). In two other photographs of *C. striata* from western North America (162423228: Santa Clara Co., California; 80206667: Klickitat Co., Washington), a similar, but not identical, hymenopteran was visiting the plant. These insects had an orange prothorax and may be braconid wasps. We did not observe any other credible pollinators in the iNaturalist photographs. Occasional small insects such as aphids were seen on some parts of the plant, but none appeared to be interacting specifically with a flower.

**Figure 2:**
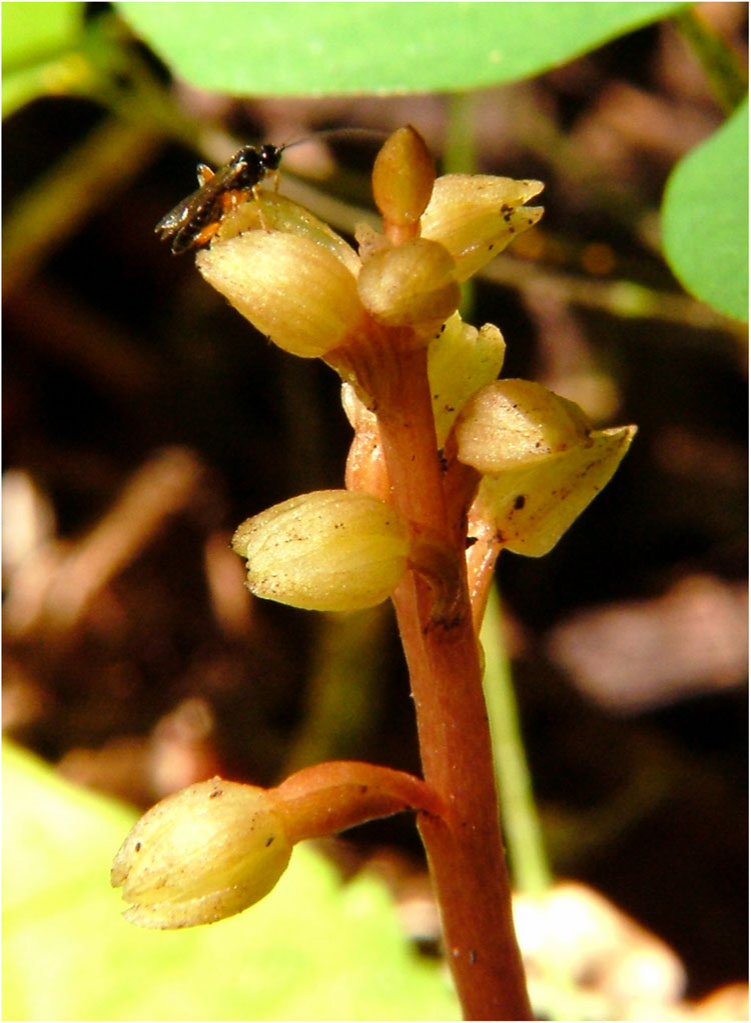
*Corallorhiza bentleyi*. Raceme with wasp on a flower (Monroe Co., West Virginia). Photograph by Charles Garratt.

We also were provided a photograph of the related *C. bentleyi* from its type locality in Monroe County, West Virginia, that has a small and very similar insect visiting it (Figure 2).

### Comparative floral morphology

In our comparison of the morphology of flowers of *C. striata* with those of its sister group, the *C. trifida* clade, the structure of the labellum notably differed. In the *C. trifida* clade, the labellum is relatively thin‐textured and planar to the margins, with small side lobes in some species (e.g., *C. maculata*; Figure 1B). Two narrow lamellae are present on the adaxial surface toward the base of the labellum. The labellum is held facing outward to downward but is not notably mobile. It is white in most species, most often with spotting. In the *C. striata* clade, the labellum is notably thickened; the lateral margins are curved upward, creating a boat‐shaped (navicular) morphology (Figure 1A). The lamellae are thickened and largely fused, creating a lobed callus near the base of the labellum. In *C. striata* var. *striata* in particular, the labellum hangs downward from the flower and is hinged by a narrow claw (Figure 1C), resulting in relatively free movement, even with a small amount of wind (video in Appendix S2).

The second notable difference between the two clades is the absence of a mentum in the *C. striata* clade. Formed from the bases of the lateral sepals and adnate to the ovary, the mentum protrudes from the ovary and is usually present in members of the *C. trifida* clade. It ranges from relatively prominent in *C. macrantha* and *C. mertensiana* to more minute in *C. odontorhiza* and *C. wisteriana*. Nectar is presumably secreted in the mentum, so the functional implication of this difference is the absence of nectar as a pollinator reward in the *C. striata* clade, consistent with the lack of nectar in capillary probes.

Other notable differences between *C. striata* var. *striata* flowers and those in the *C. trifida* clade concern coloration. All species of *Corallorhiza* have at least subtle striping on their sepals and lateral petals because 1–5 veins are present in these perianth parts. In members of the *C. trifida* clade, these veins are usually inconspicuous, not being highlighted by differential coloration, but are instead dominated by the background coloring of the perianth part and any spotting that may be present. In contrast, the common name of *C. striata* (“Striped Coralroot”) is apt, because the striping of the perianth is very pronounced; the venation is highlighted by three corresponding stripes of darker color on the background of the sepals and petals (Figure 1B).

Another differential coloration aspect concerns whether and how purplish or reddish spotting is present. In the *C. trifida* clade, spotting often occurs on the labellum, the lateral petals and the column, with less on the sepals. In the *C. striata* clade, spotting is not present on the perianth segments, and only to a limited extent on the column. It is the pattern of spotting on the column that is distinctive in *C. striata*. The spotting is limited to the base of the column and appears always to include a prominent red patch in the center of the otherwise yellowish column (Figure 1A), although additional, smaller lateral spots may also be present. Our observations of this pattern have been mainly in *C. striata* var. *striata*, but examination of photographs suggests that it also is present in *C. striata* var. *vreelandii* and among populations in the Sierra Nevada (California, USA) and Coast Ranges/Cascades (California, Oregon, USA), at least in the normal red color forms. We do not know to what extent this pattern of spotting extends to *C. involuta* and *C. bentleyi*. *Corallorhiza involuta* and *C. bentleyi* have darker reddish flowers than *C. striata* and the striping and any spotting present are much less prominent; the higher incidence of fruiting documented in var. *vreelandii* (Barrett et al., 2022), and high fruit set and tendency toward cleistogamy in *C. involuta* and *C. bentleyi* suggest that these taxa may be predominately selfing. In the *C. trifida* clade, the spotting on the column is scattered, comprising many or few small red spots; the spotting present on the perianth segments, including the labellum, also has no obvious pattern. The exception to this is the Mexican *C. bulbosa*, where the spots on the labellum are minute and copious; spotting on the basal half of the lateral petals and columns is also minute, which may suggest a distinct pollination strategy in this species.

Color patterning of the labellum also differs between the *C. trifida* and *C. striata* clades. In the *C. trifida* clade, the labellum has a white base color and small purplish spots may be present; larger blotches of color may be present in *C. mertensiana* but in no apparent pattern. In the *C. striata* clade, there is no spotting, but there is a consistent dark red line from the callus (which also may be red) to the apex, flanked by two patches of a pale yellow base color where the red is absent (Figure 1A). In our examination of images posted to iNaturalist and our own photographs, this pattern is remarkably consistent, although the length of the non‐red flanking patches varies.

Color forms occur in all commonly observed species of *Corallorhiza*, in which red‐purplish coloration (presumably due to anthocyanins) is absent, resulting in flowers with yellow perianth segments except for the labellum, which is white in the *C. trifida* clade; in *C. striata*, loss of red pigmentation results in a wholly pale yellow perianth, including the labellum. Partial color morphs also occur in many species in which intermediate coloration may be present, even in a single population. Color variation can be seen in Figure 1E, where each vertical pair of flowers comes from a different plant in the same population. The red spot at the base of the column is visible in all of these flowers. Spotting appears to be genetically controlled independently from other anthocyanin coloration in *Corallorhiza*, since otherwise normally colored flowers can be unspotted.

## DISCUSSION

### *Corallorhiza striata* as a pseudocopulatory species

*Pimpla* (*Coccygomimus*) *pedalis* was reported as a pollinator of *C. striata* var. *striata* by Freudenstein (1997) but was not investigated further until our recent studies. Townes and Townes (1960) illustrated the distribution of this species in the United States and Canada, which corresponds well with that of *C. striata* (sensu lato), although reports in the western part of the United States were sparse at that time. Although we have seen the wasp on a number of occasions visiting *C. striata* populations, some extended observations (several hours) of plants with fresh flowers in Michigan and in Wisconsin resulted in no wasp sightings, suggesting that time of day, environmental conditions, or age of the flowers could be factors, especially with respect to emission of any fragrance.

Of the four criteria that Phillips et al. (2014) listed as definitive for sexual deception, attempted copulation appears to be met here. The forward curvature of the metasoma by the visiting male insect is consistent with attempted mating behavior, as illustrated for fungus gnats on *Lepanthes* (Blanco and Barboza, 2005) and thynnine wasps on *Chiloglottis* (de Jager and Peakall, 2016); presumably the male is seeking to grasp a female metasoma with its claspers as was also depicted for other ichneumonids by Vinson (1972) and Shaw et al. (2021). Phillips et al. (2014) listed five additional criteria that are indicative of but not definitive for sexual deception. The *C. striata* system meets at least four of these—(1) males only: our close examination of 41 wasps that had visited *C. striata* revealed only males; (2) food reward absent: adult ichneumonids do consume nectar (Quicke, 2015), but our probing of flowers revealed no nectar and the morphology of the flower (with no mentum in *C. striata*) provides no place for nectar to accumulate; (3) highly specific: our sampling of *C. striata* visitors revealed only *Pimpla pedalis*, and examination of over 6000 iNaturalist records revealed only this species (and two possible braconid individuals) as potential pollinators; (4) presence of chemical attractants: our experiment with *C. striata* racemes under black mesh demonstrated attraction of male wasps in the absence of visual cues. The fifth criterion, insectiform floral structure, can be difficult to evaluate from a human perspective. Although the *C. striata* labellum does not appear as obviously insectiform as some other sexually deceptive orchids (such as *Chilogottis*), it is distinctly different from the labellum morphology of other *Corallorhiza* flowers, suggesting a different pollination strategy. In particular, the fused lamellae in the center of the labellum and distinctive and consistent spotting pattern on the base of the column may be tactile/visual cues that mimic a female wasp. The consistent color patterning of the labellum with a red stripe down the center flanked by two lighter patches could signal a wasp metasoma, whereas the callus could represent a head or mesosoma (thorax). Overall, the structural and behavioral evidence leads us to conclude that *C. striata* var. *striata* (and perhaps other varieties of this species) is engaging in a pseudocopulatory pollination strategy with *Pimpla pedalis*.

Although most instances of pseudocopulatory pollination are known from orchids, the syndrome is still rare in that group—only ca. 340 of an estimated 30,000 species of orchids (1.1%) are known to use this strategy (Gaskett, 2011; Ackerman et al., 2023). The syndrome is also not distributed evenly among major orchid groups, being known only from Orchidoideae and Epidendroideae. Greater than 99% of non‐*Lepanthes* cases are from Orchidoideae, primarily from Australasia (Diurideae) and the Mediterranean region (*Ophrys*). In the New World, reports have previously been limited to South and Central America, those primarily from Epidendroideae (e.g., *Trichoceros, Mormolyca, Lepanthes*), although *Geoblasta* from Orchidoideae has also been shown to use this strategy (Ciotek et al., 2006). Blanco and Barboza (2005) studied the behavior of *Bradysia* fungus gnats interacting with *Lepanthes glicensteinii* Luer and showed that males are deceived via a pseudocopulation strategy. They suggested that because most *Lepanthes* have a similar floral structure, the majority of the genus (currently with over 1200 accepted species; POWO, 2025) probably engages in a similar pseudocopulatory deception, which would enormously increase the number of epidendroid orchids engaged in such deception. It has subsequently been demonstrated in 10 of the 1200 species (Ackerman et al., 2023).

With respect to the insects involved in sexual deception, they may be bees, wasps, dipterans, or rarely coleopterans (Gaskett, 2011). Overall, the most commonly sexually deceived insect groups are thynnine wasps in the Australasian diurids and andrenid bees in *Ophrys*. An ichneumonid wasp was shown to be the pollinator in at least four species of Australian *Cryptostylis* (Coleman, 1927, 1930; Weinstein et al., 2016; Bohman et al., 2019); *Corallorhiza* is now the second genus and the first epidendroid known to target an ichneumonid wasp for sexual deception. *Corallorhiza striata* is also somewhat unusual among pseudocopulatory species in having not just a pollinium with or without pollinium‐derived stalks (caudicles) but a more complex pollinarium comprising pollinia and a cellular stipe. Such pollinaria are essentially limited to “vandoid” epidendroids (Rasmussen, 1986; Freudenstein and Rasmussen, 1999), meaning that of all orchid genera shown to engage in sexual deception, only *Trichoceros*, *Mormolyca*, and *Corallorhiza* have the more complex pollinaria.

*Corallorhiza striata* is widespread in North America, occurring from southern Mexico to British Columbia and east to the Atlantic (Newfoundland) in northern regions. With such a broad distribution, it is perhaps not surprising that morphological variation occurs. Freudenstein (1997) recognized three varieties (*striata*, *vreelandii*, *involuta*) based largely on flower size, with smaller flowers occurring in the southwestern part of the species’ distribution. Freudenstein (1999) described a new, related species (*C. bentleyi*) from Virginia and West Virginia, while Barrett and Freudenstein (2011) reinstated species status for *C. involuta* and showed that it is sister to *C. bentleyi*. Barrett et al. (2022) revealed four subgroups within *C. striata* (two of which correspond to vars. *striata* and *vreelandii*) and concluded they are best recognized at the varietal level. The current study focuses primarily on the largest‐flowered entity, *C. striata* var. *striata*, which occurs across the northern United States and Canada (Freudenstein, 1997).

Our experience from the field and from examination of herbarium specimens is that plants of *C. striata* var. *striata* often have only limited fruit set—sometimes only one or two per plant or none—whereas species such as *C. maculata*, which have a delayed‐selfing mechanism (Catling, 1983), usually have nearly all flowers setting fruit on a plant. *Corallorhiza striata* var. *vreelandii*, from the southwestern United States and Mexico, tends to have a less open floral morphology, with the perianth being somewhat connivent over the column and labellum, which could suggest a tendency toward self‐pollination. In fact, Barrett et al. (2022) found a significant difference between var. *vreelandii* and var. *striata* in the proportion of fruit set documented by herbarium specimens (greater in the former). The fact that we observed *P. pedalis* visiting flowers of *C. striata* var. *vreelandii* in New Mexico could indicate either that the wasp also provides significant pollination services to this variety or that the plants depend more on self‐pollination but are still effective at attracting wasps, perhaps resulting in only occasional successful pollen transfer. Even less is known about the pollination of the other two species in the *C. striata* clade: *C. involuta* and *C. bentleyi*. Both of these species have small flowers and more muted coloration with less prominent stripes than *C. striata*. *Corallorhiza bentleyi* is variable in how open the flowers are; some individuals appear to be essentially cleistogamous, as were the first specimens on which the species description was based (Freudenstein, 1999) and the photograph in Figure 2 is from that original population; the insect is visiting these flowers even though they are essentially cleistogamous. Although it is not possible to rule out that this was a chance visit, it is a male wasp, possibly a bit smaller than typical *Pimpla pedalis* but otherwise similar and could be there in response to an olfactory cue. Subsequent discoveries of additional populations of *C. bentleyi* revealed plants with more open flowers, although we have observed that ovaries are swelling by the time of anthesis even when flowers open, suggesting the species may be largely self‐pollinating. Téllez‐Baños et al. (2023) reported a coleopteran visitor to the small‐flowered *C. striata* var. *vreelandii* in Mexico but did not observe pollinaria attached to the insect.

### Double deception

It might be novel to think of pseudocopulatory pollination, or any other deceit pollination, as a type of parasitism. Definitions of parasitism vary somewhat, perhaps due to an early focus on parasitic animals that are morphologically reduced and physically attached to their hosts. Some more recent frameworks provide for a broader scope of species‐specific relationships in which one participant incurs a cost while the other incurs a benefit to qualify (e.g., Olano et al., 2011). The pseudocopulatory syndrome has been argued to be an instance of parasitism of the insect by the plant by van der Cingel (2001) and Vereecken (2009) because at minimum it results in a nonproductive cost in energy by the insect and at worst the wasteful dispensing of gametes. In this sense, it is similar to other behavioral manipulation strategies that are considered parasitism (e.g., brood parasitism).

As a vegetative parasite completely dependent on fungi for carbon metabolites, *C. striata* is specific for 1 or 2 species of ectomycorrhizal *Tomentella* (recently merged with *Thelephora*; Kõljalg et al., 2024) as hosts (*T. fuscocinerea* (Pers.) Donk, *T. patagonica* Kuhar & Rajchenb.; Barrett and Freudenstein, 2011; Barrett et al., 2022). Such narrow specificity on fungal hosts is typical for leafless orchids that are primarily heterotrophic when mature, most of which parasitize ectomycorrhizal fungi (Merckx et al., 2013; Lee et al., 2015). The orchid parasitizes an established mutualism between a vascular plant and the fungus; this relationship has been called “cheating” or “epiparasitism” because the orchid appears to be deriving benefit at the cost of the ectomycorrhizal partnership (Taylor and Bruns, 1997).

Although *C. striata* is the first example of a species with sexual deception as a pollination strategy that is also fully mycoheterotrophic, it is not the first deceit‐pollinated fully mycoheterotrophic species reported. Additional fully mycoheterotrophic orchids that deceive insect pollinators in other ways include species of *Gastrodia*—*G. similis* Bosser in Madagascar, the flowers of which mimic rotting fruit and attract a single drosophilid fly species (Martos et al., 2015), and *G. pubilabiata* Y. Sawa, which similarly targets *Drosophila*, apparently mimicking decaying fungi (Suetsugu, 2018). A case of Batesian mimicry has been found in the recently described *Danxiaorchis yangii* Bo Y. Yang & Bo Li, a species with striking yellow flowers that has been shown to mimic *Lysimachia* flowers (Luo et al., 2021) and use the *Dufourea* spp. bees (and perhaps others) that the *Lysimachia* attracts for its own pollination. Pollination of the other two species of *Danxiaorchis*, which have similar coloration, has not yet been reported. Finally, a non‐orchid full mycoheterotroph, *Thismia tentaculata* K. Larsen & Aver., was reported by Guo et al. (2019) to use a single species of the arbuscular mycorrhizal *Rhizophagus* as the trophic host and a single species of fungus gnat that visits the trap‐like flower, presumably to lay eggs on the fungus‐appearing surface. The fungal hosts for the *Danxiaorchis* and *Gastrodia* species are not yet known.

Partially mycoheterotrophic orchids (which in this case are leafless but still somewhat green and retain some photosynthetic activity) that also engage in narrowly specific deceptive pollination include *Cryptostylis hunteriana* Nicholls; it appears to target a single species of *Tulasnella* for carbon and is pollinated by ichneumonids via sexual deception (Arifin et al., 2022). *Eulophia zollingeri* J.J. Sm. is also not fully mycoheterotrophic, engaging in some photosynthesis although leafless (Suetsugu et al., 2024), but targets the single fungal family Psathyrellaceae and was found to be pollinated through food deception by the halictid *Nomia viridicinctula* Cockerell where it was studied in China (Zhang et al., 2015), although the species extends to Australia. *Cymbidium macrorhizon* Lindl. uses Sebacinales (Ogura‐Tsujita et al., 2012) for carbon and Suetsugu (2015) reported honeybees visiting the nonrewarding flowers.

Most of the aforementioned taxa are relatively limited in distribution or their distribution is poorly known (i.e., many *Gastrodia* species). *Eulophia zollingeri* may be the most widespread, occurring from southern Japan south to Australia and west to Bhutan. Although *Corallorhiza striata* var. *striata* is widespread across North America, it is uncommon over much of its distribution. To the extent that numbers of extant individuals and extent of geographic range can be used as a measure of evolutionary success, these species stand out.

Many metazoan parasites have indirect life cycles and sequential hosts, so they are dependent on more than one organism in their lifetime. Trematodes, for example, commonly have at least two hosts, one usually a snail; one trematode [*Halipegus ovocaudatus* (Vulpian)] has been shown to have four sequential obligate hosts (Kechemir, 1978). Hence, an organism parasitizing hosts from multiple species is not unusual. However, the plant species under discussion here are deceiving more than one species in a single life stage and for different specific needs (reproductive vs. trophic).

The evolutionary implications of a species that is narrowly specialized on others for both its trophic and reproductive needs are intriguing. Bidartondo (2005, p. 345) suggested that “no successful plant lineage would be expected to cheat both mycorrhizal fungi (by failing to provide photosynthates) and deceive insect pollinators (by failing to provide nectar or other rewards) due to the evolutionary instability inherent to specializing on two lineages” (a concept also developed by Waterman and Bidartondo [2008] and Waterman et al. [2013]). He argued that plants with narrow fungal specificity would likely be pollination generalists and vice versa. Highly specialized organisms, including parasites, often have been considered evolutionary “dead ends” because they are expected to be constrained by their specializations, limiting their potential for response to changing conditions (Price, 1980). If highly specialized species are more constrained and less resilient to change, having this kind of constraint on both the vegetative and reproductive axes could be especially limiting and perhaps lead to a higher probability of extinction. Here we have several species that are doing just that—specializing on different hosts for energy and reproduction and occupying a different point not on the continuum shown by Waterman et al. (2008; Figure 2).

The improbability of a double, highly specialized, non‐rewarding scenario argued by Bidartondo seems logical, but the fact that it does exist provides a useful window into this unusual condition. There are some points that should be considered when interpreting this situation, however. First, although the idea of specialization‐as‐constraint appears reasonable, studies that have assessed this hypothesis empirically have increasingly found little support for it (e.g., Flessa et al., 1975; Day et al., 2016; Zenil‐Ferguson et al., 2023), and angiosperm flowers in particular can, at least in some cases, revert to a more generalized pollination syndrome or shift to self‐pollination (Barrett, 2013). Second, the idea that parasitism is somehow expected to result in a negative outcome has also been challenged. Recent studies have argued that many host–parasite situations have no clear negative effects on host fitness (Hasik et al., 2023) or can even have positive effects (Weinersmith and Earley, 2016), and many parasite lineages are quite old (Weinstein and Kuris, 2016), suggesting that they are successful at least in the sense of persisting. Third, the fact that many sequentially multihost systems exist argues that such seemingly risky scenarios can be successful. It is unclear if the temporal dissociation of host usage seen with sequential hosts should be any less probable than simultaneous host usage.

Perhaps a major factor in determining whether a parasitism can persist long‐term is the cost incurred by the host, something that is difficult to assess empirically in mycoheterotrophic systems (Bidartondo, 2005). If that cost is significant, one would expect strong selection on the host to minimize the interaction, leading to a clear “arms race” (Dawkins and Krebs, 1979) in which parasite and host sequentially evolve strategies and counter‐strategies. If the cost to the hosts is high for both parasitisms in the double parasite system, the probability of the system persisting should be even lower. The fact that such double situations do occur may be evidence that the costs to the hosts on both the vegetative and reproductive axes are relatively low or that these species are “robust” to exploitation (Brunton‐Martin et al., 2022). Indeed, haplodiploid pollinators such as wasps can still maintain populations even when sexual reproduction is depressed (Brunton‐Martin et al., 2021).

It is also possible that the rare existence of such double parasites is due simply to the relative rarity of the two parasitic conditions they have and that there is no particular interaction effect among them. Focusing just on orchids, only about 333/30,000 (1.11%) species are fully mycoheterotrophic and about 1540/30,000 (5.13%) have presumed pseudocopulatory pollination (including *Lepanthes*). The probability of these conditions existing in a single species by chance if the conditions are independent is thus 0.00057; the actual known number of seven species that have both of these conditions (if we also include the highly specific partially mycoheterotrophic double deceivers) divided by 30,000 equals 0.00023, which is only slightly less than the calculated probability. Of course, the individual instances of these two types of parasitisms in orchids are not phylogenetically independent, since many monophyletic groups of varying size are characterized by these attributes; focusing on the number of times these parasitisms have evolved would make the joint probability of possessing both even smaller. On the other hand, it may be the persistence of each individual species as a test of its evolutionary viability that is more significant in this comparison than the number of times such parasitisms have evolved.

Examples such as *C. striata* and the other double deceivers reveal another instance of the way in which full mycoheterotrophs present unique evolutionary experiments and scenarios. Barrett and Davis (2012) argued for their use in studying plastid genome function and change under reduced selection for photosynthetic function; here we see that the system extends to providing instances of simultaneous specialized deception for nutrition and reproduction. This situation, that had been predicted to be unlikely, has now been observed in six to seven independent mycoheterotroph lineages, two of which are widespread, revealing that such a combination of deceit strategies not only can exist, but also can be successful.

## CONCLUSIONS

*Corallorhiza striata* is the first example of a pseudocopulatory orchid from North America, and *Corallorhiza* is only the second genus known to deceive an ichneumonid wasp for pollination. It is unusual in being the first leafless, fungal‐parasitic plant to be also sexually deceptive. Only four other species (three orchids) of full mycoheterotrophs and three partial mycoheterotrophs are currently known to engage simultaneously in fungal and floral (food reward) deception. Thus, although rare, simultaneous multiple deception can be a successful strategy. Such parasitic plants provide unusual opportunities to study evolutionary and ecological phenomena from new perspectives.

## AUTHOR CONTRIBUTIONS

J.V.F. conceived the study, collected material, and performed experiments in the field, analyzed morphology, and wrote the initial draft. C.F.B. conceived the study, collected material in the field, analyzed morphology, and contributed to writing.

## CONFLICT OF INTEREST STATEMENT

John V. Freudenstein is an Associate Editor of the *American Journal of Botany* but took no part in the peer‐review and decision‐making processes for this paper.

## Supporting information


**Appendix S1.** Video of *Pimpla pedalis* visiting *Corallorhiza striata* var. *striata*.

**Appendix S2.** Video of *Corallorhiza striata* var. *striata* flowers in moving air to illustrate mobility of the labellum.

## Data Availability

Online observations of *C. striata* used for this study are posted on iNaturalist (inaturalist.org).
